# Sedentary behavior and type 2 diabetes in community-dwelling middle-aged and older adults: indirect cross-sectional correlation of sarcopenic obesity

**DOI:** 10.3389/fendo.2026.1813608

**Published:** 2026-08-14

**Authors:** Wenwen Zhao, Mingjie Meng, Yiheng Geng, Ruyue Qi, Jinjia Zhang, Min Zhang, Meijing Song, Rongying Wang

**Affiliations:** 1 General Practice Department, `The Second Hospital of Hebei Medical University, Shijiazhuang, Hebei, China; 2 Department of Cardiology, Division III, The Second Hospital of Hebei Medical University, Shijiazhuang, Hebei, China; 3 School of Public Health, Hebei Medical University, Shijiazhuang, Hebei, China; 4 General Practice Department, Community Service Centre of Lianmeng Subdistrict Office, Shijiazhuang, Hebei, China

**Keywords:** sedentary behavior, sarcopenic obesity, statistical mediation, middle-aged and elderly population, age-specific

## Abstract

**Background:**

Type 2 diabetes mellitus (T2DM) represents a major public health challenge among middle-aged and older populations worldwide. The underlying mechanisms linking sedentary behavior to T2DM remain incompletely understood. Sarcopenic obesity (SO), a phenotype defined by concurrent low muscle mass and excess adiposity, may represent a key intermediate correlate linking sedentary behavior with T2DM in cross-sectional observations. This study aimed to explore the indirect cross-sectional correlation of SO in the correlation between sedentary behavior and T2DM and to clarify age- and sex-specific characteristics of this association.

**Methods:**

A cross-sectional study design was employed, consecutively enrolling 2, 480 community-dwelling middle-aged and older individuals (≥35 years) undergoing physical examinations. Daily sedentary time was assessed using the Sedentary Behavior Questionnaire (SBQ). Body composition was measured with the InBody 770 bioelectrical impedance analyzer. Logistic regression combined with bias-corrected, non-parametric Bootstrap methods validated the statistical mediation, with sensitivity analyses stratified by sex, age, and the age-sex interaction.

**Results:**

In the overall cohort, the statistical mediation of SO was significant (without covariates: mean ACME = 0.105, 95% CI: 0.078–0.130, proportion of the statistical indirect correlation = 22.43%; with covariates: mean ACME = 0.029, 95% CI: 0.013–0.043, proportion of the statistical indirect correlation = 14.97%;both *P* < 0.01). Stratified analyses revealed significant heterogeneity in the statistical mediation: the share of the statistical indirect correlation was higher in women (22.68%) than in men (9.08%), and significantly higher in those aged ≥65 years (19.14%) than in those aged <65 years (8.93%). In the age-sex interaction analysis, the statistical mediation was strongest in the ≥65-year female subgroup (mean ACME = 0.121, proportion of the statistical indirect correlation = 47.79%, *P* < 0.01), while the statistical mediation were not significant in the <65-year male (*P* = 0.20) and <65-year female (*P* = 0.11) subgroups.

**Conclusion:**

Sedentary behavior is significantly associated with type 2 diabetes among community-dwelling middle-aged and older adults, and SO shows a significant statistical mediation in the cross-sectional correlation between sedentary behavior and T2DM. This statistical mediation shows distinct population differences and is most prominent among women aged 65 years or older.

## 1 Introduction

Diabetes has become one of the foremost public health challenges facing middle-aged and older populations worldwide. Data from the 11th edition of the IDF Diabetes Atlas, released by the International Diabetes Federation (IDF) in 2025, indicate that by 2024, the global adult diabetes population aged 20–79 years reached 589 million, with type 2 diabetes mellitus (T2DM) accounting for over 90% (approximately 530 million), and individuals aged 45 years and older constituting over 60% of the total. Annual global health-related expenditures attributable to diabetes total USD 1.1 trillion, with healthcare systems bearing 67% of direct costs for diagnosis, treatment, and complication management. The remaining burden stems from indirect costs, such as lost productivity, posing severe challenges to healthcare resource allocation and sustainable development worldwide.

With shifts in modern lifestyles, sedentary behavior (defined as accumulating 7–9 hours of daily sitting) has been confirmed as a modifiable associated factor for T2DM. A dose-response meta-analysis incorporating 58 observational studies from 48 publications found that each additional hour of total daily sedentary time in adults was associated with a 5% higher prevalence of T2DM. Specifically, each extra hour of television viewing was associated with an 8% higher prevalence of T2DM. A prospective study from Harvard University involving approximately 70, 000 women demonstrated a strong correlation between sedentary behavior and T2DM. Specifically, each additional 2 hours of daily television viewing was associated with a 14% higher prevalence of T2DM, and individuals with moderate-to-high sedentary time (≥ 3 hours daily) had a 50%–80% higher prevalence than those with low sedentary time (1).

Cross-sectional correlational evidence linking sedentary behavior to T2DM-related metabolic abnormalities has revealed complex biological correlates, although definitive temporal pathways remain unconfirmed. Existing studies have reported associative links between sedentary behavior and insulin resistance and glucose metabolism disorders, including inhibition of insulin-mediated glucose uptake in skeletal muscle, promotion of ectopic visceral fat accumulation, and downregulation of muscle protein synthesis efficiency through suppression of the mTOR signaling pathway (2). Sarcopenic obesity (SO), a distinct phenotype characterized by concurrent muscle mass reduction and elevated body fat percentage, may serve as a key intermediate factor in the correlation between the two conditions. SO is defined by diminished skeletal muscle mass/function and increased body fat percentage. This condition affects approximately 11% of older adults globally, with significantly higher prevalence rates observed in individuals with T2DM, reaching approximately 20% (3). Asian populations exhibit relatively lower baseline muscle mass reserves due to population-specific body composition characteristics. Concurrently, dietary shifts toward higher refined carbohydrate intake amplify this vulnerability. The combined effects of these factors contribute to a relatively higher prevalence of SO (4). The 2025 Asian Working Group for Sarcopenia (AWGS) confirms a strong association between SO and T2DM. The prevalence of sarcopenia reaches 20.5% among patients with T2DM. By disrupting crosstalk between muscle and other organs, SO coexists with more severe metabolic abnormalities in cross-sectional samples, and, compared with simple obesity or sarcopenia, SO is correlated with a higher prevalence of T2DM (5).

This cross-sectional study utilized community health examination data, using logistic regression with Bootstrap sampling to analyze statistical mediation. Results were validated by stratifying participants by sex and age to clarify the indirect cross-sectional correlation of SO and identify population-specific patterns.

## 2 Methods

### 2.1 Study design and participants

A cross-sectional study design was employed. Continuous sampling was conducted from 1 April 1 to 30 June 30 2025, among individuals undergoing health examinations at the Community Service Center in Lianmeng Subdistrict, Xinhua District, Shijiazhuang City, Hebei Province, China. Participants aged ≥35 years undergoing health examinations were included in the study.

Inclusion criteria: 1. Ability to complete the SBQ questionnaire, InBody 770 body composition analysis, and laboratory tests; 2. Complete clinical records; and 3. Informed consent and voluntary participation in the study.

Exclusion criteria: 1. Severe acute or chronic diseases (e.g., active malignancy, end-stage renal disease, decompensated cirrhosis, or New York Heart Association (NYHA) Class III-IV heart failure); 2. Presence of major mobility impairments (e.g., amputation, severe arthritis, or bedridden status); 3. Major surgery within the past 3 months or current use of medications known to significantly affect muscle metabolism (e.g., systemic corticosteroids or androgen deprivation therapy); 4. Pregnant or lactating; 5. Unable to undergo reliable assessment due to severe cognitive impairment or psychiatric disorders.

### 2.2 Primary study variables

#### 2.2.1 Sedentary behavior

The Sedentary Behavior Questionnaire (SBQ) was used for assessment. Trained investigators guided participants in completing the questionnaire, which measured cumulative daily sedentary time (e.g., watching TV, computer use, reading, and commuting) in hours per day (h/d). This data was analyzed as quantitative information. High sedentary behavior was clinically defined as ≥8 h/day, with <8 h/day classified as low sedentary behavior (6, 7).

#### 2.2.2 Intermediate correlate: SO

Body composition was measured using the InBody 770 bioelectrical impedance analyzer (InBody 770, Korea). Measurement protocol: Participants fasted for ≥8 h before measurement. They removed all metal objects and heavy clothing, stood barefoot on the device’s electrode plates, held the electrode handles with both hands, and maintained an upright, stationary posture. The device automatically performed whole-body impedance measurements, providing data including limb skeletal muscle mass and body fat percentage. The limb skeletal muscle mass index (ASMI) was calculated as follows: ASMI = skeletal muscle mass of limbs/height²(kg/m²).

SO diagnostic criteria: Based on the 2019 Asian Consensus Criteria for Sarcopenia (8) and the Chinese Guidelines for the Diagnosis and Treatment of Obesity (2024 Edition) (9):

Reduced muscle mass: ASMI <7.0 kg/m² for men and ASMI < 5.7 kg/m²for women.

Obesity: body fat percentage ≥25% for men and ≥30% for women.

SO: concurrent fulfillment of the above criteria for both low muscle mass and obesity.

#### 2.2.3 Outcome variable: type 2 diabetes

T2DM was diagnosed according to the Chinese Guidelines for the Prevention and Treatment of Type 2 Diabetes (2024 Edition) (10) combined with laboratory test results. Diagnostic criteria included: 1. Fasting blood glucose (FBG) ≥7.0 mmol/L;2. Postprandial 2-h blood glucose (2hPBG) ≥ 11.1 mmol/L;3. Glycated hemoglobin (HbA1c) ≥ 6.5%;4. A previous diagnosis of T2DM with current hypoglycemic treatment (including oral agents or insulin) or a confirmed history of type 2 diabetes mellitus. Meeting any of the above criteria was considered diagnostic of T2DM.

#### 2.2.4 Confounding factors

Potential confounding factors included family history of type 2 diabetes (first-degree relatives with type 2 diabetes);lifestyle factors, including smoking history (yes: cumulative smoking ≥100 cigarettes or regular smoking ≥6 months; no), alcohol consumption history (yes: drinking ≥1 time per week), high-salt diet (daily salt intake ≥5g), and high-oil diet (daily cooking oil intake ≥30g); and clinical indicators, including systolic blood pressure (SBP, mmHg), diastolic blood pressure (DBP, mmHg), total cholesterol (TC, mmol/L), triglycerides (TG, mmol/L), and low-density lipoprotein cholesterol (LDL-C, mmol/L).

### 2.3 Data collection

All participants completed standardized questionnaires and underwent anthropometric measurements and laboratory testing. Blood pressure was measured using an electronic sphygmomanometer on the right brachial artery after at least 5 min of rest.

Laboratory test data: Fasting blood glucose (FBG), 2-hour postprandial blood glucose (2hPBG), glycated hemoglobin (HbA1c), total cholesterol (TC), triglycerides (TG), and low-density lipoprotein cholesterol (LDL-C) were extracted from the physical examination database of the Community Health Service Center in Lianmeng Subdistrict, Xinhua District, Shijiazhuang City. Instrumentation and models: FBG and 2hPBG were measured using the Siemens ADVIA 2400 fully automated biochemical analyzer; HbA1c was measure using the Roche Cobas c513 fully automated glycated hemoglobin analyzer; TC, TG, and LDL-C were analyzed using the Beckman Coulter AU5800 fully automated biochemical analysis system. All blood samples were centrifuged within 1 h and tested within 1–3 h in the community service center laboratory according to standardized procedures.

### 2.4 Statistical analysis

Statistical analysis was performed using SPSS 26.0 and R 4.5.1 (mediation package). The significance level was set at α = 0.05 (two-tailed), with *P* < 0.01 indicating statistically significant differences.

#### 2.4.1 Descriptive statistics

Continuous variables normally distributed were expressed as mean ± standard deviation (x ± s); non-normally distributed variables were presented as median (P25, P75).

Categorical variables were reported as counts (proportions).

#### 2.4.2 Intergroup comparisons

Continuous variables were compared using independent samples t-tests (for normal distribution) or Mann-Whitney U tests (for non-normal distribution). For categorical variables, Pearson’s χ² test or Fisher’s exact test (when the theoretical frequency <5) was used.

#### 2.4.3 Statistical indirect correlation decomposition

A three-step regression approach was used, with three binary logistic regression models constructed:

Model 1 (total effect): T2DM was the dependent variable, sedentary time was the independent variable, and all confounders were included. Model 2 (exposure-intermediate correlate association): SO was the dependent variable, sedentary behavior was the independent variable, and the same confounders were incorporated. Model 3 (direct effect + statistical mediation): T2DM was the dependent variable, with sedentary time and SO simultaneously included.

This indirect cross-sectional correlation decomposition was conducted under three standard theoretical assumptions for statistical correlation partitioning frameworks in observational epidemiology (11): complete elimination of unmeasured confounding across exposure, intermediate correlate, and outcome (12); absence of reverse causal relationships between all study variables; and (13) perfect error-free measurement of SO and T2DM status.

We adjusted for a comprehensive panel of demographic, lifestyle, and clinical covariates to mitigate measurable confounding bias. However, several critical limitations prevent full satisfaction of these assumptions within our cross-sectional design. First, residual unmeasured confounders (e.g., unrecorded dietary quality, regular resistance training, and inflammatory biomarkers) cannot be fully ruled out. Second, cross-sectional data cannot rule out reverse associations: insulin resistance and T2DM may concurrently reduce physical activity and accelerate muscle loss, which would violate the no-reverse-causality premise. Third, self-reported sedentary time and BIA-derived body composition measurements contain inherent random measurement error, violating the assumption of perfectly reliable variable quantification.

Accordingly, all decomposed indirect and direct associations should be interpreted only as statistical correlation partitioning, rather than formal causal mediation estimates. The analytical framework follows the classical statistical correlation partitioning framework for calculating the indirect correlation component value recommended in epidemiological and public health literature.

Bootstrap validation: The bias-corrected non-parametric Bootstrap method (5,000 resamples) was used to calculate the indirect correlational component value and its 95% bias-corrected confidence interval (95% CI). If the 95% CI did not include 0, the indirect correlational component was considered significant. The indirect correlational component contribution ratio was calculated as follows: Indirect correlational component contribution ratio = (Total effect value - Direct effect value)/Total effect value×100%.

Stratified analysis: Repeated mediation analyses were conducted by stratifying for sex, age (≥65 years vs. <65 years), and age-sex interaction (≥65 years old men, ≥65 years old women, <65 years old men, and <65 years old women) to explore population heterogeneity.

Sensitivity analysis: Result stability was evaluated using stratified validation methods, specifically: repeat indirect cross-sectional correlation decomposition stratified by sex; repeat indirect cross-sectional correlation decomposition stratified by age (≥65 years vs. <65 years); and repeat indirect cross-sectional correlation decomposition stratified by age-sex interaction (≥65years old men, ≥65years old women, <65years old men, and <65years old women). The robustness of the findings was assessed by examining the fluctuation range of the OR values for the statistical mediation across each stratum (≤15% indicating stability) and whether the 95% confidence intervals consistently excluded zero (indicating significant stratification). Importantly, causal mediation requires longitudinal or interventional data to establish temporality, which our cross-sectional dataset cannot provide.

## 3 Results

### 3.1 Baseline characteristics of study participants

This study included 2, 480 participants, comprising 1, 293 men (52.14%) and 1, 187 women (47.86%). The T2DM group consisted of 358 participants (14.44%) and the SO group included 183 participants (7.38%). Compared with the non-T2DM group, the T2DM group showed significantly higher proportions of age, family history of type 2 diabetes, high-salt/high-oil diet, and smoking history, in addition to significantly higher levels of SBP, DBP, FBG, 2hFBG, HbA1c (%), TC, TG, LDL-C, sedentary time, sedentary behavior, and SO prevalence (all *P* < 0.01). Differences in sex were statistically significant (χ² = 7.13, *P* < 0.01), while differences in body mass index (BMI) were not statistically significant (Z = –1.60, *P* = 0.11), and differences in alcohol consumption history were not statistically significant (χ² = 2.45, *P* = 0.12). The overall prevalence of sarcopenic obesity was 7.38%; the prevalence was higher among women than among men and was significantly higher among adults aged 65 years and older than among younger adults. In the age-sex interaction subgroups, the prevalence of sarcopenic obesity was highest among women aged 65 years and older (**Table 1**).

**Table 1:** Baseline characteristics of community-dwelling middle-aged and older adults according to T2DM status.

Baseline characteristic	T2DM		χ ^2^/Z value	*P*-value
	Yes n (%)	No n (%)		
Sex			7.13	<0.01
Men	210 (8.47%)	1083 (43.66%)		
Women	148 (5.97%)	1039 (41.90%)		
Age (years)	63.50 (57.75, 71.25)	59.00 (49.00, 68.00)	-8.33	<0.01
BMI (kg/m^2^)	25.40 (23.30, 27.83)	25.90 (23.60, 28.50)	-1.60	0.11
Family history of T2DM			580.48	<0.01
Yes	240 (9.68%)	255 (10.28%)		
No	118 (4.76%)	1867 (75.28%)		
Sedentary time (hours/day)	8.40 (7.60, 8.90)	6.10 (4.60, 6.80)	-26.76	<0.01
Sedentary behavior			649.27	<0.01
Yes	238 (9.60%)	216 (8.71%)		
No	120 (4.84%)	1906 (76.85%)		
High-oil diet			177.54	<0.01
Yes	249 (10.04%)	692 (27.90%)		
No	109 (4.40%)	1430 (57.66%)		
High-salt diet			224.39	<0.01
Yes	111 (4.48%)	125 (5.04%)		
No	247 (9.96%)	1997 (80.52%)		
Smoking history			105.05	<0.01
Yes	111 (4.48%)	230 (9.27%)		
No	247 (9.96%)	1892 (76.29%)		
Alcohol consumption history			2.45	0.12
Yes	94 (3.79%)	644 (25.97%)		
No	264 (10.65%)	1478 (59.59%)		
Sarcopenic obesity			542.61	<0.01
Yes	133 (5.36%)	50 (2.02%)		
No	225 (9.07%)	2072 (83.55%)		
SBP (mmHg)	142 (126, 146)	122 (116, 126)	-19.53	<0.01
DBP (mmHg)	93 (76, 96)	72 (68, 82)	-18.09	<0.01
FBG (mmol/L)	7.20 (6.90, 7.90)	5.20 (5.10, 5.40)	-26.09	<0.01
2hPBG (mmol/L)	11.40 (10.80, 11.83)	7.90 (7.60, 8.10)	-25.59	<0.01
HbA1c (%)	7.40 (6.98, 7.5)	5.40 (5.30, 5.50)	-25.57	<0.01
TC (mmol/L)	5.40 (5.00, 5.70)	4.60 (4.50, 4.80)	-20.66	<0.01
TG (mmol/L)	1.60 (1.20, 2.40)	1.00 (0.80, 1.10)	-21.42	<0.01
LDL-C (mmol/L)	2.00 (1.70, 2.40)	1.80 (1.40, 1.90)	-12.79	<0.01

### 3.2 Results of indirect overlap partitioning

Analysis of the total effect model (Model 1) revealed a significant positive association between sedentary behavior and T2DM (OR = 10.84, 95% CI: 7.24, 16.21). This relatively high OR value is discussed below. Without including the intermediate correlate, sedentary behavior was independently associated with T2DM in this population.

The exposure-intermediate correlate association model (Model 2) revealed that sedentary behavior was significantly associated with SO (OR = 6.47, 95% CI: 4.09, 10.23), suggesting that sedentary behavior substantially was positively associated with SO, fulfilling the prerequisite for exposure-intermediate correlate association in indirect cross-sectional correlation decomposition.

When both sedentary behavior and SO were incorporated into Model 3, both variables displayed independent positive cross-sectional correlations with T2DM. The direct correlational magnitude of sedentary behavior (OR = 8.51, 95% CI: 5.62, 12.90) was attenuated relative to the total crude correlation, and the statistical indirect correlational component involving SO reached statistical significance (OR = 5.69, 95% CI: 3.14, 10.32). This pattern reflects the statistical partitioning of cross-sectional pairwise associations within our dataset (**Table 2**).

**Table 2:** Statistical indirect correlation decomposition for sedentary behavior and T2DM.

Model type	Dependent variable	Independent variable/intermediate correlate	OR (95% CI)	*P*
Model 1				
Total effect	T2DM	Sedentary behavior	10.84 (7.24, 16.21)	<0.01
Model 2				
Exposure-intermediate correlate association	SO	Sedentary behavior	6.47 (4.09, 10.23)	<0.01
Model 3				
Combined effect	T2DM	Sedentary behavior	8.51 (5.62, 12.90)	<0.01
		SO	5.69 (3.14, 10.32)	<0.01

In the overall population, the statistical mediation of SO was significant (no covariates: mean ACME = 0.105, 95% CI: 0.078, 0.130, proportion of the statistical indirect correlation = 22.43%;with covariates: mean ACME = 0.029, 95% CI: 0.013, 0.043, proportion of the statistical indirect correlation = 14.97%;both *P* < 0.001). Covariates partially attenuated the association but did not alter the indirect cross-sectional correlation.

Stratification by sex and age revealed a higher proportion of the statistical indirect correlation in women (22.68%) than men (9.08%). The magnitude of the statistical indirect correlational pattern was significantly stronger in individuals aged ≥65 years (mean ACME = 0.056, proportion of the statistical indirect correlation = 19.14%) compared to those aged<65 years (mean ACME = 0.012, proportion of the statistical indirect correlation = 8.93%). Direct effects were significant across all stratifications (all *P* < 0.001).

The age-sex interaction analysis revealed that the strongest SO statistical mediation occurred in the ≥65-year + female subgroup (mean ACME = 0.121, proportion of the statistical indirect correlation = 47.79%, *P* < 0.01). In contrast, the < 65-year + male subgroup (mean ACME = 0.012, *P* = 0.20) and < 65-year + female subgroup (mean ACME = 0.007, *P =* 0.11) showed non-significant SO statistical mediation, with only direct effects being significant (both *P* < 0.001).

The group with the largest indirect correlational share was the ≥65-year female subgroup, where approximately half of the total cross-sectional overlap corresponds to the indirect component linked to SO (47.79%), with significant effects.

**Figure 1:**
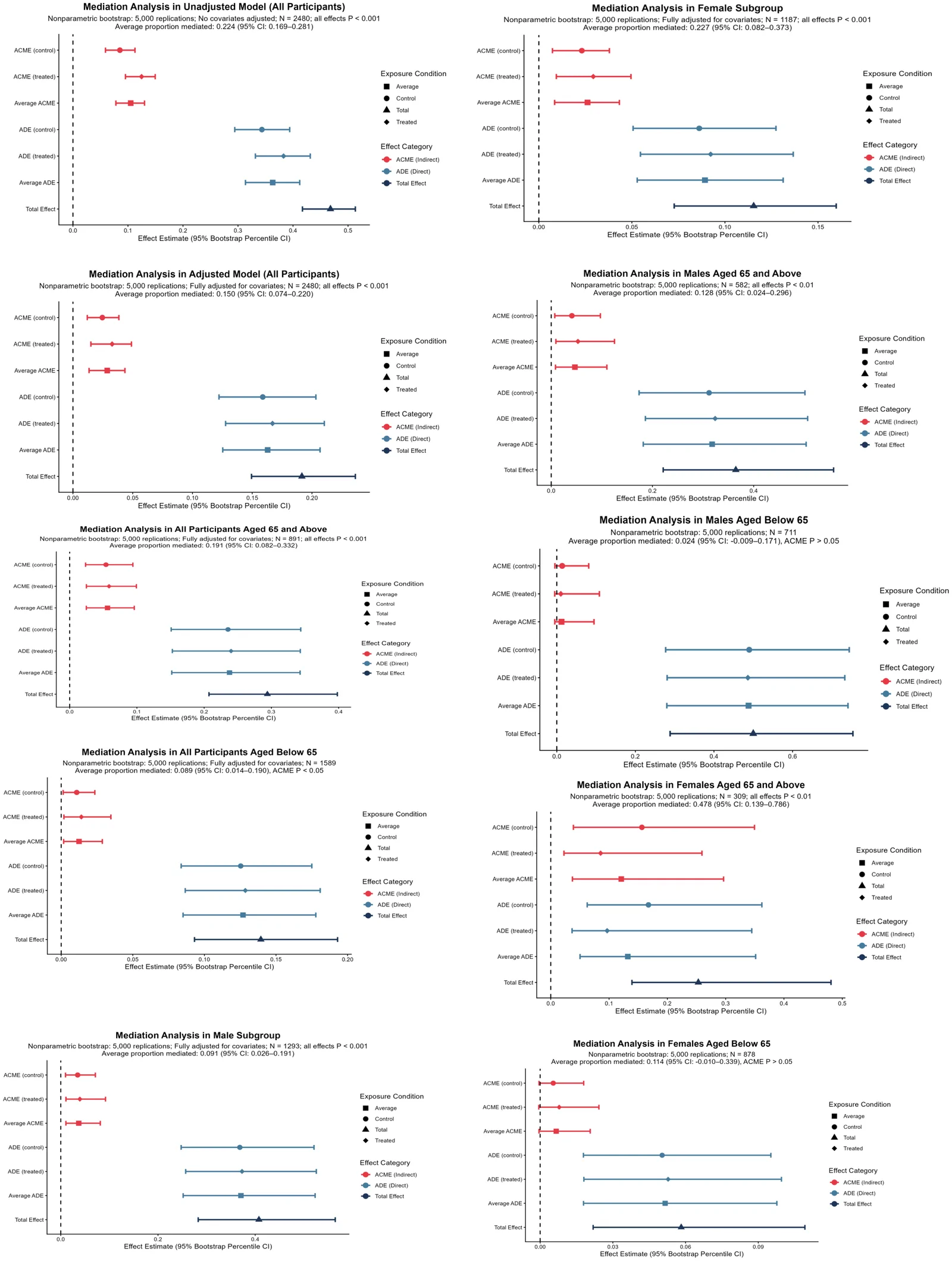
Forest plots of statistical indirect correlation patterns based on binary daily sedentary behavior.

Groups with detectable indirect overlapping correlations (*P* < 0.05) generally exhibited the following pattern: older individuals > younger individuals and women > men. The magnitude of the indirect correlational pattern decreased but remained significant after covariate adjustment. Groups with non-significant indirect overlapping correlations *(P*>0.05) included the < 65-year male and female subgroups, which had low proportions of statistical indirect correlations (2.39%–11.39%) and 95% CIs encompassing zero, indicating a non-significant indirect correlational pattern of SO among younger populations (**Figure 1**; **Table 3**).

**Table 3:** Statistical indirect correlation patterns of SO estimated using Bootstrap.

Group	ACME (95% CI)	ADE (95% CI)	Total effect(95% CI)	Average share of the statistical indirect correlation (%)(95% CI)	Sample size	*P*
Total population (without covariates)	0.105 (0.078, 0.130)	0.363 (0.314, 0.412)	0.468 (0.417, 0.513)	22.43 (16.87, 28.12)	2480	***
Total population (with covariates)	0.029 (0.013, 0.043)	0.163 (0.125, 0.207)	0.192 (0.149, 0.236)	14.97 (7.38, 21.97)	2480	***
Men	0.037 (0.011, 0.081)	0.371 (0.252, 0.523)	0.408 (0.283, 0.565)	9.08 (2.58, 19.13)	1293	***
Women	0.026 (0.008, 0.043)	0.089 (0.053, 0.131)	0.115 (0.073, 0.160)	22.68 (8.17, 37.31)	1187	***
Aged 65 years and older	0.056 (0.025, 0.096	0.238 (0.152, 0.343)	0.294 (0.207, 0.398)	19.14 (8.23, 33.18)	891	***
Aged under 65 years	0.012 (0.002, 0.029	0.127 (0.085, 0.178)	0.139 (0.093, 0.193)	8.93 (1.41, 19.01)	1589	*
Men aged 65 and older	0.047 (0.008, 0.110)	0.318 (0.182, 0.504)	0.365 (0.221, 0.558)	12.84 (2.39, 29.60)	582	**
Women aged 65 and older	0.121 (0.037, 0.297)	0.132 (0.050, 0.351)	0.253 (0.139, 0.481	47.79 (13.87, 78.64)	309	**
Men aged under 65 years	0.012 (-0.005, 0.095)	0.488 (0.280, 0.741)	0.500 (0.288, 0.754)	2.39 (-0.01, 0.17)	711	0.20
Women aged under 65 years	0.007 (-0.000, 0.021)	0.052 (0.018, 0.098	0.058 (0.022, 0.109)	11.39 (-0.01, 0.34)	878	0.11

All mediating indicators including average causal mediation effect (ACME), average direct effect (ADE), total effect, and the proportion of indirect mediating effect were calculated via two-tailed nonparametric percentile bootstrap with 5,000 resamples. Significance markers were defined as ***P<0.001, **P<0.01, *P<0.05; exact P-values were listed for non-significant mediating effects. ACME, ADE and total effect values for the association between sedentary behavior and sarcopenic obesity (SO) were rounded to three decimal places, as were the upper and lower limits of all 95% confidence intervals (95% CI). The proportion of the indirect mediating effect was reported as a percentage with two decimal places.

A sensitivity analysis was conducted by recoding daily sedentary time as a continuous variable rather than a binary classification to test the stability of our main findings.

In the fully adjusted model using continuous sedentary exposure, the proportion of the total cross-sectional correlation accounted for by SO decreased substantially from 14.97% (binary sedentary variable) to 5.80%, accompanied by a reduction in the average ACME, which declined from 0.029 (binary sedentary variable) to 0.018 when sedentary time was modeled as continuous, with the share of the statistical indirect correlation falling substantially from 14.97% to 5.80%. Although the 95% CIs of the indirect association still excluded zero and confirmed statistical significance, the marked attenuation of the share of the indirect correlation indicated that the magnitude of SO’s indirect correlational contribution was substantially overestimated when sedentary time was dichotomized.

**Figure 2:**
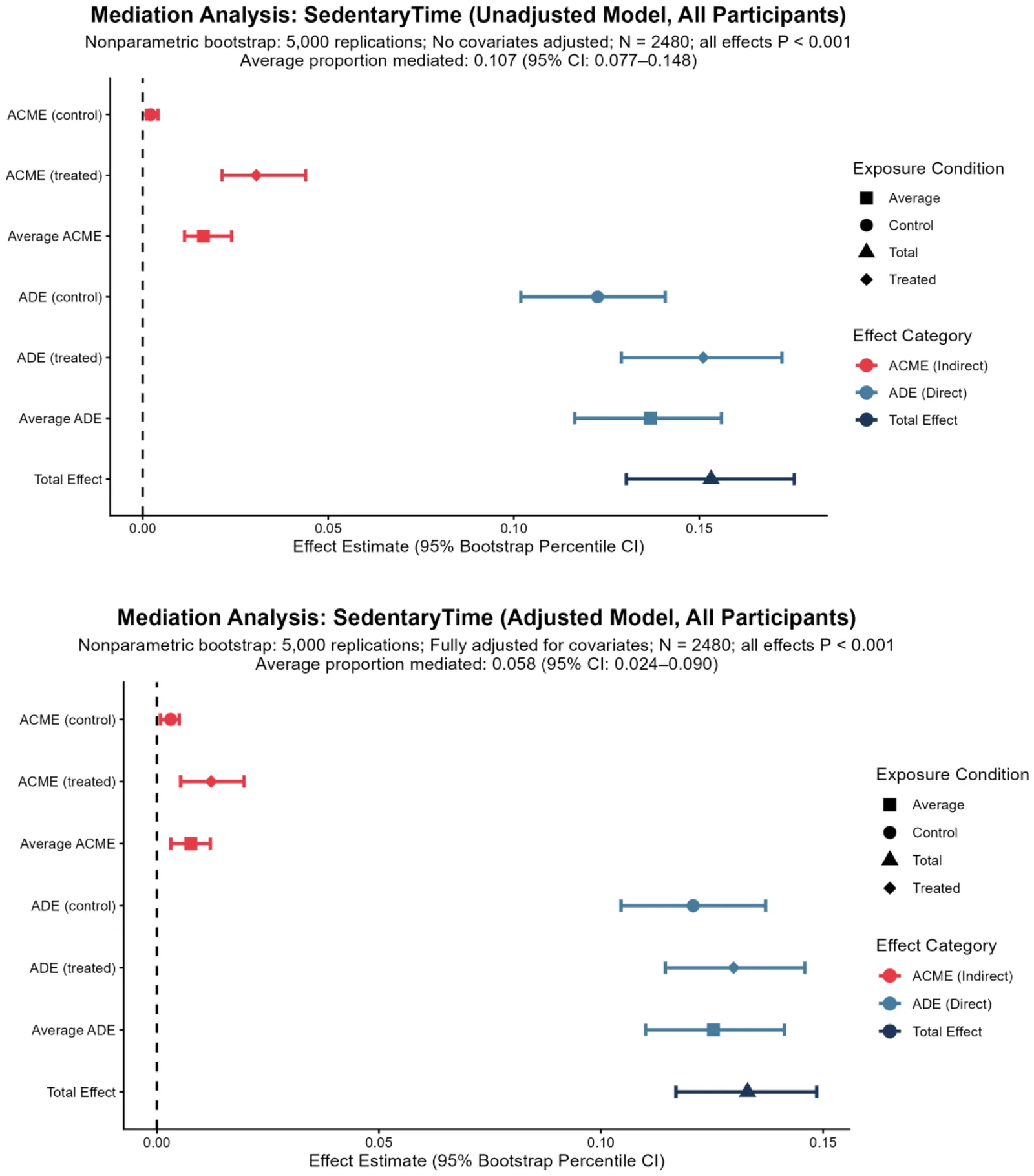
Forest plots of statistical indirect correlation patterns based on continuous daily sedentary time.

This notable decline highlights that the strength of the statistical indirect association is highly dependent on how sedentary exposure is operationalized. While the core conclusion of a detectable indirect overlapping correlation pattern remained robust across variable coding schemes, the real population-level contribution of SO to the sedentary-T2DM correlation is far weaker than the binary analysis initially suggested (**Figure 2**).

## 4 Discussion

The markedly elevated OR observed for sedentary behavior and T2DM is likely driven by the binary categorization of exposure and cross-sectional reverse correlation and should not be interpreted as a causal risk magnitude. This study used a cross-sectional design and cannot confirm causal relationships. All findings reflect only statistical associations between variables. The relatively high odds ratio (OR = 10.84) observed in this study can be explained by several factors. Dichotomization of sedentary behavior may have enlarged intergroup differences and increased the observed association strength. Residual unmeasured confounders and reverse association inherent to the cross-sectional design may also have contributed to effect overestimation, as individuals with T2DM or impaired muscle function tend to have longer sedentary time. Additionally, the homogeneous sample from a single community and characteristics of binary logistic regression may have affected model stability. Therefore, this value represents only a statistical association rather than a causal effect and should be interpreted with caution.

Among community-dwelling older adults, results from cross-sectional correlation partitioning indicated a detectable indirect overlapping correlation linking sedentary behavior and T2DM within this cross-sectional sample. The statistical mediation varied by age and sex, was significant among individuals aged 65 and older, and was strongest among older women. Sensitivity analysis was conducted by treating sitting duration (a binary variable) as a continuous variable. The results from the stratified subgroups were consistent with those of the original binary model, demonstrating the robustness of the findings. Based solely on cross-sectional correlational data from a single community cohort, targeted SO screening and sedentary reduction assessments may provide preliminary reference information; however, long-term cohort studies and intervention trials are needed to determine whether such measures provide clinical benefits for women aged ≥65 years. Longitudinal interventional research is required to verify whether such measures could alter T2DM risk. Due to the limitations of this single-community cross-sectional study, further validation through multicenter studies is warranted. Our sensitivity analysis using continuous sedentary time further refined the interpretation of the indirect association. The substantial reduction in the proportion of the indirect correlation (from approximately 15% to approximately 6%) demonstrated that binary grouping of sedentary time may amplify the apparent indirect effect of SO. Researchers should exercise caution when interpreting the proportion of indirect overlapping correlation derived from dichotomized exposure variables in cross-sectional metabolic studies. It should be noted that all results from cross-sectional correlation partitioning are interpreted under the basic assumptions of statistical indirect cross-sectional correlation decomposition frameworks for observational research. The intermediate associative role of SO is supported by previous pathological and epidemiological findings. In this study, the proportion of the indirect overlapping correlation associated with SO in the overall population was 22.43% before and 14.97% after adjusting for covariates, suggesting SO accounted for a measurable portion of the statistical indirect correlation linking sedentary behavior and T2DM within the cross-sectional data, even after controlling for confounding factors such as family history and lifestyle. This aligns with findings from Yuan et al.’s (14) Mendelian randomization study, which reported associations between sedentary behavior and metabolic disease-related outcomes, including adiposity gain, muscle loss, and chronic inflammatory markers. Based on accumulated epidemiological and preclinical correlative evidence, we summarize a tentative statistical association overlapping pairwise correlations derived solely from cross-sectional observational data: within this sample, longer sedentary time was associated with higher SO prevalence, and insulin resistance and higher T2DM rates co-occurred among participants with SO, although no confirmed chronological sequence among the three indicators can be established. Importantly, these overlapping pairwise cross-sectional correlations reflect concurrent co-occurrence only and cannot confirm temporality or any causal relationships between variables. No definitive biological causal pathway can be inferred from the present dataset. On one hand, prolonged sedentary time has been cross-sectionally associated with reduced skeletal muscle synthesis and decreased glucose uptake efficiency through inhibition of satellite cell activation and downregulation of the mTOR signaling pathway (15, 16). On the other hand, the energy imbalance caused by sedentary behavior may contribute to visceral fat accumulation, resulting in the concurrent presence of reduced muscle mass and excess adiposity with muscle decline (17). In individuals with SO, intermuscular adipose tissue (IMAT), intramuscular adipose tissue (IMCL), and visceral fat may release free fatty acids (FFA) and pro-inflammatory factors (TNF-α, IL-6), which are associated with impaired insulin signaling through lipotoxic and inflammatory pathways (18–20). Animal studies further support this associative link: mice subjected to simulated sedentary conditions exhibited impaired glucose regulation within 3 weeks, accompanied by reduced insulin receptor phosphorylation levels. SO was associated with more prominent overlapping metabolic correlations in this cross-sectional sample, and these overlapping metabolic overlaps are more prominent among participants with SO (21, 22).

Population heterogeneity analysis revealed that clear heterogeneity exists in this overlapping correlation across age and sex subgroups in statistical mediation. Age-stratified results showed a significantly higher proportion of the statistical indirect correlation (19.14%) among individuals aged ≥65 years compared with those aged <65 years (8.93%), with the latter exhibiting only marginally significant indirect correlational components. This disparity mainly arises from age-related declines in physiological reserves. Compared with younger adults, older people show a 40%–60% decline in the proliferation and differentiation capacity of muscle satellite cells. Mitochondrial dysfunction impairs skeletal muscle oxidative metabolism, and defective myoglobin synthesis accompanied by accelerated muscle protein degradation strengthens the mediating effect of sarcopenic obesity on the link between sedentary behavior and type 2 diabetes (23–25). In contrast, younger and middle-aged adults possess greater muscle reserves and metabolic flexibility, which may buffer short-term energy imbalance and muscle function suppression caused by sedentary behavior through intrinsic metabolic regulation (26–28). This corresponds to weaker overlapping cross-sectional correlations of SO, consistent with the widely observed heightened sensitivity of older adults to metabolic prevalence factors (29–31).

Sex heterogeneity was particularly pronounced among the older subgroup: the proportion of the statistical indirect correlation among women aged ≥65 years (47.79%) substantially exceeded that among men (12.84%). This difference may be partially explained by three correlational biological patterns observed in previous literature. First, men exhibit a greater tendency toward visceral fat accumulation. Male obesity is often characterized by adipocyte hypertrophy, inflammation, and fibrosis, resulting in impaired adipocyte function. Free fatty acids released directly enter the portal vein and act on the liver, inducing lipotoxicity, insulin resistance, and impaired glucose metabolism, thereby exacerbating systemic metabolic disorders. In contrast, women predominantly accumulate subcutaneous adipose tissue (SAT), which generally exhibits more stable metabolism and lower lipolytic activity. Their SO relies more heavily on muscle-adipose interactions, where this effect is more pronounced (17, 32). Second, hormonal regulation differs between sexes. Although postmenopausal women experience a significant reduction in the primary ovarian estrogen (17β-estradiol, E2), peripheral tissues such as fat and muscle can convert precursor substances into residual physiological levels of estrogen via aromatase. These residual estrogens have been cross-sectionally linked to altered insulin signaling and reduced pro-inflammatory factor release through estrogen receptor alpha (ERα) in previous observational studies (e.g., TNF-α, IL-6). This alleviates inflammation-mediated insulin resistance, thereby exerting protective effects on insulin sensitivity.³ (13) Third, prolonged sedentary time was cross-sectionally correlated with greater suppression of muscle metabolism among men, corresponding to overlapping muscle mass-related cross-sectional correlations. In women, prolonged sitting primarily influences metabolic syndrome through direct effects, with muscle mass contributing less to the observed indirect cross-sectional correlation. This may relate to the presence of light activity interruptions during prolonged sitting, resulting in less severe muscle function impairment. Among individuals younger than 65 years, the statistical mediation of SO were not significant in either sex. Sedentary behavior directly influences T2DM, potentially affecting glucose metabolism through pathways beyond SO, such as cross-sectionally correlated with disrupted skeletal muscle GLUT4-mediated glucose transport or elevated odds of simple obesity (33–35).

With rising global T2DM prevalence and accelerating population aging, precision interventions tailored to demographic characteristics are critical. Based solely on single-site cross-sectional correlational data, targeted SO screening and sedentary time evaluation may be considered for women aged ≥65 years; however, long-term cohort studies and interventional research are needed to verify whether such strategies can lower T2DM risk.

For men aged ≥65 years, although the statistical mediation of SO was weaker, the direct effect of sedentary behavior on T2DM was strong. Therefore, reducing sedentary time may represent a potential intervention target, but this is only preliminary suggestive guidance, and interventional studies are required to confirm its metabolic benefits. Furthermore, these findings support incorporating SO into T2DM prevalence assessment systems for the older adults, particularly for older women with a history of prolonged sedentary behavior. Attention to SO status may provide an important reference for population health management related to T2DM.

## 5 Conclusion

In community-dwelling older adults, SO exhibited a significant statistical mediation within the cross-sectional relationship between sedentary behavior and T2DM. This statistical mediation demonstrated strong age specificity (significant only among individuals aged ≥65 years) and sex heterogeneity within the older age subgroup (with the largest share of the statistical indirect correlational pattern observed among women aged 65 years and older). No detectable indirect overlapping correlation linked to SO was observed among participants younger than 65 years. These findings highlight the importance of age and sex in metabolic correlation assessment and provide a scientific reference for targeted population health management related to T2DM. Given the cross-sectional observational design, targeted SO screening and sedentary reduction interventions may serve as preliminary population health references for community health management; however, multicenter longitudinal trials are required to validate these suggestions, particularly among middle-aged and older adults and women aged ≥65 years. Community-based resistance training and sedentary reduction programs should be considered exploratory approaches pending validation through long-term cohort studies. Due to the single-community sampling design, the generalizability of our findings is limited. Further multicenter investigations are warranted to confirm these results. Collectively, the unmet theoretical assumptions for indirect cross-sectional correlation decomposition inherent to cross-sectional observational data further emphasize that all reported indirect associations represent correlational patterns rather than validated causal pathways.

## 6 Limitations

Several limitations of this study should be acknowledged. First, the cross-sectional design limits the interpretation of causal relationships and cannot determine the temporal order among sedentary behavior, sarcopenic obesity, and T2DM. Second, all sedentary time data were collected via self-reported questionnaires, which may introduce recall and reporting bias. Third, body composition was measured using bioelectrical impedance analysis (BIA). Although BIA is a widely used portable method, its results may be affected by hydration status, skin condition, and body posture, leading to minor measurement errors compared with the gold standard method, dual-energy X-ray absorptiometry (DXA). Fourth, participants were recruited from a single community, and potential selection bias cannot be fully excluded. To reduce measurement bias, all investigators received standardized training, and unified operating protocols were strictly implemented throughout data collection and testing. In addition, the odds ratios may have been overestimated due to dichotomous grouping, residual confounding, and reverse association. Therefore, caution is needed when interpreting these cross-sectional results. Fifth, the definition of sarcopenic obesity in this study relied solely on low appendicular skeletal muscle mass index and elevated body fat percentage, without incorporating functional muscle indicators, including grip strength, gait speed, and physical performance metrics. Several international sarcopenic obesity diagnostic frameworks integrate both muscle quantity and muscle function to identify this phenotype. Exclusion of functional assessments may restrict the comparability and external validity of our SO prevalence estimates and could potentially lead to underestimation or misclassification of participants with subtle muscle dysfunction despite preserved muscle mass. Future investigations combining both quantitative and functional muscle measures are required to replicate these correlational findings.

## Data Availability

The original contributions presented in the study are included in the article/supplementary material. Further inquiries can be directed to the corresponding author.
